# Photosynthesis in C_3_–C_4_ intermediate *Moricandia* species

**DOI:** 10.1093/jxb/erw391

**Published:** 2016-10-19

**Authors:** Urte Schlüter, Andrea Bräutigam, Udo Gowik, Michael Melzer, Pascal-Antoine Christin, Samantha Kurz, Tabea Mettler-Altmann, Andreas PM Weber

**Affiliations:** 1 Institute of Plant Biochemistry, Cluster of Excellence on Plant Sciences (CEPLAS), Heinrich Heine University, Universitätsstr. 1, Düsseldorf, Germany; 2 Institute of Plant Molecular and Developmental Biology, Cluster of Excellence on Plant Sciences (CEPLAS), Heinrich Heine University, Universitätsstr, Düsseldorf, Germany; 3 Network Analysis and Modelling, Leibniz Institute of Plant Genetics and Crop Research (IPK), OT Gatersleben, Corrensstr, Stadt Seeland, Germany; 4 Structural Cell Biology, Leibniz Institute of Plant Genetics and Crop Research (IPK), OT Gatersleben, Corrensstr, Stadt Seeland, Germany; 5 Department of Animal and Plant Sciences, University of Sheffield, Western Bank, Sheffield, UK

**Keywords:** Bundle sheath, C_3_–C_4_ intermediacy, C_4_ photosynthesis, evolution, glycine decarboxylase, *Moricandia*

## Abstract

Evolution of C_4_ photosynthesis is not distributed evenly in the plant kingdom. Particularly interesting is the situation in the Brassicaceae, because the family contains no C_4_ species, but several C_3_–C_4_ intermediates, mainly in the genus *Moricandia*. Investigation of leaf anatomy, gas exchange parameters, the metabolome, and the transcriptome of two C_3_–C_4_ intermediate *Moricandia* species, *M. arvensis* and *M. suffruticosa*, and their close C_3_ relative *M. moricandioides* enabled us to unravel the specific C_3_–C_4_ characteristics in these *Moricandia* lines. Reduced CO_2_ compensation points in these lines were accompanied by anatomical adjustments, such as centripetal concentration of organelles in the bundle sheath, and metabolic adjustments, such as the balancing of C and N metabolism between mesophyll and bundle sheath cells by multiple pathways. Evolution from C_3_ to C_3_–C_4_ intermediacy was probably facilitated first by loss of one copy of the glycine decarboxylase P-protein, followed by dominant activity of a bundle sheath-specific element in its promoter. In contrast to recent models, installation of the C_3_–C_4_ pathway was not accompanied by enhanced activity of the C_4_ cycle. Our results indicate that metabolic limitations connected to N metabolism or anatomical limitations connected to vein density could have constrained evolution of C_4_ in *Moricandia*.

## Teaser

Analysis of the genus *Moricandia*, which contains C_3_ and C_3_–C_4_ intermediate plants, reveals potential environmental and anatomical constraints to the evolution of C_4_ photosynthesis.

## Introduction

C_4_ plants evolved in warm, open, and often arid regions, where the C_4_ concentrating mechanism leads to enhanced photosynthetic carbon fixation efficiency (Sage, 2004). In most C_4_ species, this is achieved by upstream CO_2_ fixation through phospho*enol*pyruvate carboxylase (PEPC) in the mesophyll (MS) and transport of the synthesised C_4_ metabolites to the bundle sheath (BS) cells (Hatch, 1987). Decarboxylation of C_4_ metabolites in the BS cells increases CO_2_ concentration around Ribulose-1,5 bisphosphate carboxylase/oxygenase (Rubisco), thus promoting the carboxylase reaction while reducing photorespiration (Hatch and Slack, 1970; Bowes *et al.*, 1971; Hatch, 1987). Division of photosynthetic biochemistry between two different cell types requires anatomical adaptation including high vein density, reduction of MS tissue, and enlarged, chloroplast-rich BS cells (Dengler *et al.*, 1994; McKown and Dengler, 2007; Christin *et al.*, 2013). Despite its complexity, the C_4_ trait evolved independently more than 60 times in flowering plants (Sage *et al.*, 2012; Sage, 2016). Soon after the discovery of C_4_ photosynthesis, it became apparent that transition from C_3_ to C_4_ photosynthesis could not have been realised in one giant step, but more likely evolved via a series of transitory states (Kennedy and Laetsch, 1974). Potential C_3_–C_4_ intermediates were identified by their CO_2_ compensation point, which lay between the values of C_3_ and C_4_ species, as well as some C_4_-like anatomical features in the BS cells (Kennedy and Laetsch, 1974; Krenzer *et al.*, 1975). The Brassicaceae species *Moricandia arvensis* was among the first species classified as a potential C_3_–C_4_ intermediate (Krenzer *et al.*, 1975).

The genus *Moricandia* consists of eight accepted species (www.theplantlist.org), all originating from Mediterranean or Saharo-Sindian areas (Tahir and Watts, 2010). Based on CO_2_ compensation points, *Moricandia* includes C_3_ species (*M. moricandioides*, *M. foetida*, and *M. foleyi*) as well as C_3_–C_4_ intermediates (*M. arvensis*, *M. suffruticosa*, *M. nitens*, *M. spinosa*, and *M. sinaica*; Brown and Hattersley, 1989; Apel *et al.*, 1997). Besides a low CO_2_ compensation point, the C_3_–C_4_ candidates exhibit lower sensitivity to O_2_ (Holaday *et al.*, 1982) and high incorporation of ^14^C into glycine and serine (Holaday and Chollet, 1983; Hunt *et al.*, 1987). The BS area per cell profile is increased as well as the number of chloroplasts, mitochondria, and peroxisomes in the BS (Holaday and Chollet, 1983; Hunt *et al.*, 1987). In contrast to C_4_ species, these potential intermediates possess a C_3_-like δ^13^C signature, C_3_-like Rubisco kinetics (Bauwe and Apel, 1979; Holbrook *et al.*, 1985), and low activities of typical C_4_ enzymes such as PEPC, pyruvate phosphate dikinase (PPDK), NADP malic enzyme (NADP-ME), NAD-ME, and phospho*enol*pyruvate carboxykinase (PEPCK) (Holaday *et al.*, 1981; Holaday and Chollet, 1983).

Rawsthorne and colleagues showed that the P-subunit of the glycine decarboxylase multi-enzyme system (GLDP) is exclusively localised to the BS of the leaf of *M. arvensis* (Rawsthorne *et al.*, 1988*a*, *b*; Rylott *et al.*, 1998), while all other enzymes of the photorespiratory or photosynthetic pathways, such as the L, H, and T subunits of GLD, serine hydroxymethyltransferase (SHMT), glycolate oxidase (GOX), and Rubisco, are found in MS as well as BS tissues (Rawsthorne *et al.*, 1988*b*, Morgan *et al.*, 1993). These findings led to the first experimentally verified model of photosynthesis in C_3_–C_4_ intermediates (Rawsthorne, 1992). The interruption of the photorespiratory cycle in the MS caused by the absence of the functioning GLD/SHMT complex leads to an accumulation of glycine and its diffusion to the BS cells. There, its decarboxylation creates a local CO_2_ enrichment, thus increasing the carboxylation activity of Rubisco in the BS and therefore reducing the CO_2_ compensation point of the leaf (Rawsthorne, 1992). This process is also named the glycine shuttle, photorespiratory CO_2_ pump, or C_2_ photosynthesis (Sage *et al.*, 2014).

Species with C_3_–C_4_ intermediate characteristics have been identified in diverse groups of plants (Krenzer *et al.*, 1975; Rajendrudu *et al.*, 1986; Moore *et al.*, 1987; Hylton *et al.*, 1988; Apel *et al.*, 1997; Muhaidat *et al.*, 2011; Sage *et al.*, 2011*b*; Wen and Zhang, 2015; Khoshravesh *et al.*, 2016). Phylogenetic studies have shown that many of these C_3_–C_4_ plants are closely related to C_4_ siblings, and it is therefore likely that intermediates served as transitory states on the evolutionary path from C_3_ to C_4_ (McKown *et al.*, 2005; Christin *et al.*, 2011*a*; Fisher *et al.*, 2015; Lyu *et al.*, 2015). The possibility that C_3_–C_4_ intermediates bridge the evolutionary gap between C_3_ and C_4_ states has also recently been supported by different computational modelling approaches (Heckmann *et al.*, 2013; Williams *et al.*, 2013; Mallmann *et al.*, 2014; Brautigam and Gowik, 2016). Experimental as well as computational models predict that under favourable genetic and anatomic pre-conditions, the shift of GLD to the BS is a decisive step for installation of the glycine shuttle and the transition from C_3_ to C_3_–C_4_ photosynthesis. Because the GLD/SHMT reaction in the BS releases not only CO_2_ but also NH_3_, rebalancing of the N metabolism between the two cell types is required (Mallmann *et al.*, 2014). For re-balancing of N metabolism, the current model suggests additional metabolite shuttles, e.g. glutamate–2-oxoglutarate, alanine–pyruvate, and aspartate–malate. Parts of these shuttles and the associated biochemical enzymes also play an important role in C_4_ photosynthesis. Existing C_4_ enzymes and transporters can create a primordial C_4_ cycle in the C_3_–C_4_ background on which selection can act as long as selective pressure for efficient C assimilation persists (Aubry *et al.*, 2011; Mallmann *et al.*, 2014; Brautigam and Gowik, 2016). In essence, altered GLD localisation is predicted to initiate a smooth path to C_4_ (Heckmann *et al.*, 2013; Brautigam and Gowik, 2016; Schluter and Weber, 2016). In support of this model, over 90% of plant lineages with C_3_–C_4_ intermediates also contain C_4_ species (Mallmann *et al.*, 2014).

The model, however, fails to explain the presence of evolutionary stable C_3_–C_4_ intermediates. The situation in the Brassicaceae is therefore particularly interesting; to date no *sensu stricto* C_4_ species have been identified in this family, but it contains multiple lines of C_3_–C_4_ intermediates, including members of the genus *Moricandia* (Hylton *et al.*, 1988), *Diplotaxis tenuifolia* (Apel *et al.*, 1997; Ueno *et al.*, 2006), and *Brassica gravinae* (Ueno, 2011). In this current study, the C_3_–C_4_ metabolism in the genus *Moricandia* was investigated in more detail by simultaneous analyses of phylogeny, leaf anatomy, gas exchange, and metabolite and transcript patterns in closely related C_3_ (*M. moricandioides*) and C_3_–C_4_ species (*M. arvensis* line MOR1 and *M. suffruticosa*). The data were used for testing hypotheses related to C_4_ evolution or lack thereof: (i) phylogenetic patterns of GLDP explain the evolution of intermediacy in just one specific branch of the Brassicaceae; (ii) metabolic differences between C_3_ and intermediate species relate to the N-shuttle; and (iii) differences with the genus *Flaveria*, which evolved full C_4_, indicate where *Moricandia* species might be restricted in evolution towards C_4_.

## Material and methods

### Plant cultivation

**Fig. 1.**
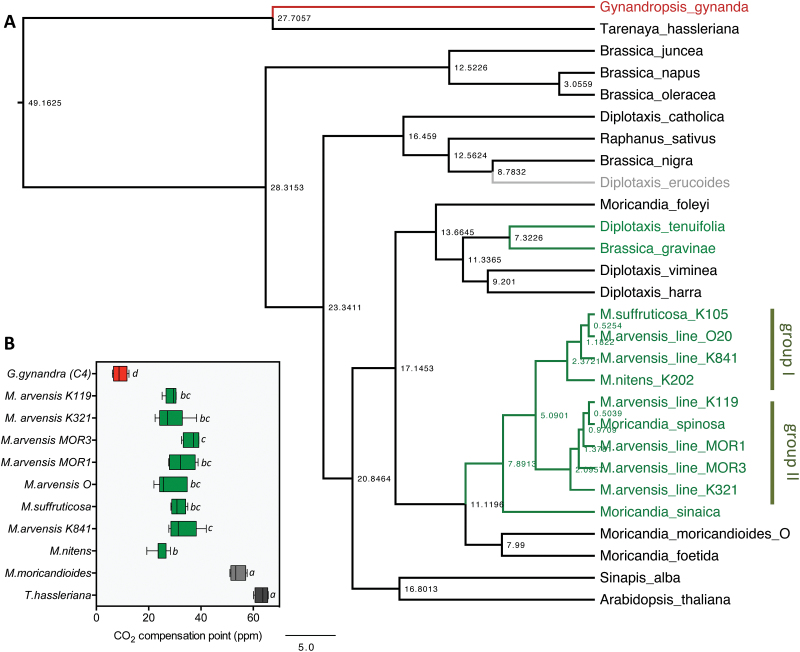
C_3_-C_4_ intermediate species in the Brassicaceae. (A) Time-calibrated phylogeny of the Brassicaceae species selected for this study. The *Moricandia* species build one branch of the tree with an early separation of the C_3_ species *M. moricandioides* and *M. foetida*, and the C_3_–C_4_ intermediate species *M. suffruticosa*, *M spinosa*, *M. sinaica*, *M. nitens*, and different *M. arvensis* lines. Additional independently established C_3_–C_4_ intermediate species are *Diplotaxis tenuifolia* and *Brassica gravinae*. The closest C_4_ relative *G. gynandra* belongs to the Cleomaceae. C_3_ species are in black; C_3_–C_4_ intermediate species in green, with gray for the potential intermediate *D. erucoides*; C_4_ species in red. The scale bar is 5 Ma. (B) CO_2_ compensation points determined from *A*-*C*_i_ curves in C_3_ and C_3_–C_4_*Moricandia* lines compared with C_3_ and C_4_ species from the Cleomaceae. Significant differences were determined by ANOVA followed by Tukey’s HSD multiple comparison test with alpha ≤0.01.

Seeds for different *Moricandia* lines were obtained from botanic gardens and seed collections (*Moricandia moricandioides*, 04-0393-10-00 from Osnabrück Botanic Gardens; *M. arvensis*, line 12-0020-10-00 from Osnabrück Botanic Gardens, lines 0119708, 0000321, 0084187 from the Royal Botanic Gardens in Kew, lines MOR1 and MOR3 from IPK Gatersleben; *M. suffruticosa*, line 0105433 from the Royal Botanic Gardens in Kew; *M. nitens*, 0209858 from the Royal Botanic Gardens in Kew). Seeds were vapour-sterilised by incubation in a desiccator together with a freshly prepared mix of 100 ml 13% Na-hypochloride with 3 ml of 37% HCl for 2 h. The sterilised seeds were then germinated on plates containing 0.22% (w/v) Murashige Skoog medium, 50 mM MES pH 5.7 and 0.8% (w/v) Agar. After about a week, seedlings were transferred to pots containing a mixture of sand and soil (BP substrate, Klasmann & Deilmann GmbH, Germany) at a ratio of 1:2. In the first experiment, testing all the *Moricandia* lines, plants were grown in a greenhouse (Heinrich Heine University, Düsseldorf) in September 2013, where they received natural light ranging from 300 and 600 µmol s^−1^ m^−2^. For our more detailed studies of C_3_–C_4_ intermediate metabolism in *Moricandia*, we chose one species from each *Moricandia* C_3_–C_4_ subgroup presented in Fig. 1A (*M. suffruticosa* from group I and *M. arvensis* line MOR1 from group II) and compared them to their C_3_ relative *M. moricandioides*. For all following experiments, plants were cultivated in a climate chamber (CLF Mobilux Growbanks, Wertingen, Germany) under 12-h day conditions with 23/20 °C day/night temperatures. The plants were exposed to ambient CO_2_ concentrations and irradiance at plant level was ~200 µmol s^−1^ m^−2^. All experiments were conducted before the transition to the reproductive state. Mature leaf material was harvested from *M. moricandioides*, *M. arvensis* (line MOR1), and *M. suffruticosa* ~2 h after the start of the light period by flash-freezing in liquid nitrogen. The material was homogenised into a fine powder by grinding in liquid nitrogen. The material was stored at –80 °C and used for analysis of elements, metabolites, transcripts, and proteins.

### Phylogeny

Relationships between *Moricandia* species and their closest relatives were determined using sequences from the ITS nuclear region. DNA was extracted from all the *Moricandia* lines available in our study, *Diplotaxis tenuifolia* (line ‘Wilde Rauke’, origin: N.L. Chrestensen Erfurter Samen-und Pflanzenzucht GmbH), and *D. viminea* (line GB.31066, origin: Rijk Zwaan Distribution B.V., Netherlands). The ITS sequences were amplified and sequenced using the primers ITS1 (O’Kane *et al.*, 1996) and ITS4 (White *et al.*, 1990). Additional ITS sequences were retrieved from the NCBI database (see Supplementary Table S1 at *JXB* online). The sequences were aligned with Muscle (Edgar, 2004), and the alignment was used to infer a time-calibrated phylogeny following a relaxed molecular clock approach, as implemented in Beast (Drummond and Rambaut, 2007). The analysis was run for 10 000 000 generations, sampling a tree every 1000 generations under a GTR+G+I substitution model, a log-normal relaxed clock, and a Yule speciation prior. The tree was rooted by forcing the monophyly of both the outgroup (the two ‘*Cleome*’ species) and the ingroup (Brassicaceae). The tree was calibrated by setting the age of the crown of Brassicaceae with a normal distribution with a mean of 29.3 Ma and a standard deviation of 3.0, based on estimates from markers across nuclear genomes (Christin *et al.*, 2014). The convergence of the analysis and adequacy of the burn-in period were verified using Tracer (Drummond and Rambaut, 2007). Medians of node ages for tree samples after a burn-in period of 1 000 000 generations were mapped on the maximum-credibility tree using Treeannotator (Drummond and Rambaut, 2007).

GLDP-specific mRNA sequences were retrieved from online databases (see Supplementary Table S2). GLDP-specific coding sequences for *Moricandia* and *Diplotaxis* lines were obtained from the assembly of next-generation sequencing reads produced in our own lab (see below). The phylogenetic tree was constructed with the help of the Phylogeny.fr webtool (http://phylogeny.lirmm.fr) in the default mode consisting of alignment by Muscle, G-block building, maximum-likelihood tree generation by PhyML, and visualisation by TreeDyn.

### Plant anatomy

Vein density measurements were done on the top third of fully grown rosette leaves. The leaf material was cleared in an acetic acid:ethanol mix (1:3) overnight followed by staining of cell walls in 5% safranin O in ethanol, and de-staining in 70% ethanol. Pictures were taken using a Nikon Eclipse Ti-U microscope equipped with a ProgRes MF camera (Jenoptik, Jena, Germany), at 4× magnification. At least six leaves were analysed for vein density per line, always with five pictures measured and averaged per leaf using ImageJ open software (https://imagej.nih.gov/ij/).

For histological and ultrastructural analysis 2-mm^2^ sections from mature rosette leaves were used for combined conventional and microwave-proceeded fixation, dehydration, and resin embedding in a PELCO BioWave®34700-230 (Ted Pella, Inc., Redding CA, USA) as described in Supplementary Table S3. Semi-thin sections with a thickness of approximately 2.5 µm were mounted on slides and stained for 2 min with 1 % methylene blue / 1% Azur II in 1% aqueous borax at 60 °C prior to light microscopical examination in a Zeiss Axio Imager M2 microscope (Carl Zeiss Microscopy GmbH, Göttingen, Germany). Ultra-thin sections with a thickness of approximately 70 nm were cut with a diamond knife, transferred onto TEM-grids and contrasted in a LEICA EM STAIN (Leica Microsystems, Vienna, Austria) with uranyl acetate and Reynolds’ lead citrate prior to analysis using a Tecnai Sphera G2 transmission electron microscope (FEI, Eindhoven, Netherlands) at 120 kV.

### Photosynthetic gas exchange

Mature rosette leaves were used for gas exchange measurements with a LICOR 6400XT (LI-COR Biosciences, Lincoln, USA). The settings were a flow of 300 µmol s^−1^, light of 1500 µmol m^−2^ s^−1^, leaf temperature of 25 °C, and the vapour pressure deficit was kept below 1.5 kPa. Initial analysis of the *Moricandia* lines and comparison with the related C_3_ plants *Brassica oleraceae* and *Tarenaya hassleriana*, the C_3_–C_4_ intermediate *Diplotaxis tenuifolia*, and the C_4_ plant *Gynandropsis gynandra* were done with *A*-*C*_i_ curves, with measurments at 400, 50 100, 200, 400, 800, 1200, and 1800 ppm CO_2_. Significance of the differences in the CO_2_ compensation points between lines was tested using a one-way ANOVA followed by a post-hoc Tukey’s multiple comparison test.

More detailed *A*-*C*_i_ curves were measured on the selected species *M. moricandioides*, *M. arvensis* MOR1, and *M. suffruticosa*. After acclimation, an *A*-*C*_i_ curve was determined starting at a CO_2_ concentration (*C*_a_) of 400 ppm, then going down to 20 ppm in nine steps, then going back to 400 ppm and raising the CO_2_ concentration stepwise up to 1800 ppm. Measurements at the lowest six CO_2_ concentrations were used to extract the CO_2_ compensation point and the initial slope of the graph corresponding to the carboxylation efficiency. Maximal assimilation was determined at CO_2_ concentrations of 1600 to 1800 ppm. At least six plants were measured per line, and statistical significance between values for the different species was evaluated using Student’s *t*-test.

### Metabolite and element analysis

The homogenized leaf material was extracted for metabolite analysis by gas chromatography-mass spectrometry (GC-MS) according to Fiehn *et al.* (2000) using a 7200 GC-QTOF (Agilent, Santa Clara, USA). Data analysis was conducted with the Mass Hunter Software (Agilent, Santa Clara, USA). For relative quantification, all metabolite peak areas were normalized to the peak area of an internal standard of ribitol added prior to extraction. The same homogenised leaf material was used for determination of δ^13^C and CN ratios. After lyophilisation, the material was analysed using an Isoprime 100 isotope ratio mass spectrometer coupled to an ISOTOPE cube elemental analyzer (both from Elementar, Hanau, Germany) according to Gowik *et al.* (2011). Measurements were always done on ten biological replicates. Statistical significance was analysed using Student’s *t*-test.

### Transcript analysis

Total RNA was extracted from the homogenized leaf material using the GeneMatrix Universal RNA purification kit (Roboklon GmbH, Berlin, Germany). The RNA was treated with DNase for a few seconds only and quality controlled on a Bioanalyzer 2100 (Agilent, Santa Clara, USA). Subsequently, mRNA purification and adapter ligation was performed with the TruSeq RNA Sample Preparation Kit (Illumina, San Diego, USA) using 1 μg of total RNA. After a second quality control on the Bioanalyzer, samples were sent to Beckmann Coulters Genomics (Danvers, MA, USA) and sequenced on a HiSeq2500 sequencer (Illumina, San Diego, CA) as single-end 100-bp reads. Three to four biological replicates were used per *Moricandia* species with between 13 and 17 million reads per sample. The reads were aligned to a minimal set of coding sequences of the TAIR 10 release of the Arabidopsis genome (http://www.arabidopsis.org/) using BLAT (Kent, 2002) in protein space. The best BLAT hit for each read was determined by (1) the lowest *e*-value and (2) the highest bit score (Brautigam *et al.*, 2011). Raw read counts were transformed to reads per million (rpm) to normalize for the number of reads available at each sampling stage. Cross-species mapping takes advantage of the completeness and annotation of the Arabidopsis genome. In all samples, 80 to 86% of reads mapped to the reference genome. Comparison of the transcript pattern between species was performed with the edgeR tool (Robinson *et al.*, 2010) in R (www.R-project.org) using the Benjamini–Hochberg false discovery test with a cut-off at false discovery rate (FDR) ≤0.01 for significant differences. The agriGO webtool was employed for gene ontology (GO) term analysis (Du *et al.*, 2010). The transcriptome data was deposited at the GEO repository (www.ncbi.nlm.nih.gov/geo/) under the accession number GSE87343.

For construction of the specific *Moricandia* transcript assemblies, one sample per line was sequenced as pair-end 150-bp reads on an Illumina HiSeq2000 platform at the BMFZ (Biologisch-Medizinisches Forschungszentrum) of the Heinrich Heine University (Düsseldorf, Germany). The resulting reads were trimmed using the trimmomatic tool (Bolger *et al.*, 2014) followed by assembly using Trinity (Haas *et al.*, 2013), which yielded between 68 000 and 91 000 contigs. Sequences for transcripts from *D. tenuifolia*, *D. viminea*, and *D. muralis* were obtained following the same protocol.

### PEPC activity

Total soluble proteins were extracted from the homogenised leaf material in 50 mM Hepes-KOH pH 7.5, 5 mM MgCl_2_, 2 mM DTT, 1 mM EDTA, 0.5% Triton-X-100, 0.1% ß-mercaptoethanol. For the PEPC assay, 20 µl of the extract were mixed with assay buffer consisting of 100 mM Tricine-KOH pH 8.0, 5 mM MgCl_2_, 2 mM DTT, 1 mM KHCO_3_, 0.2 mM NADH, 5 mM glucose-6-phosphate, 2 U ml^−1^ malate dehydrogenase in a microtiter plate (Ashton *et al.*, 1990). The reaction was started after addition of phospho*enol*pyruvate to a final concentration of 5 mM in the assay. Absorbance at 340 nm was measured with a Synergy HT microplate reader (BioTek Instruments, USA). Protein content of the solutions was determined with the Bradford assay (Quick Start Bradford Protein Assay kit, BioRad). The ratio between fresh and dry weight of the leaf material was determined by weighing a second mature leaf from the same plants before and after drying it at 70 °C overnight.

## Results

### Occurrence of C_3_–C_4_ intermediates in the Brassicaceae

The phylogenetic relationships between all the *Moricandia* accessions available in our study were investigated by sequencing their ITS region and in comparison with data available in the NCBI database (Fig. 1A). We aimed at testing whether the C_3_–C_4_ character evolved independently or in one single event in different *Moricandia* lines. With the exception of *M. foleyi*, all *Moricandia* species formed a monophyletic group in the phylogenetic tree (Fig. 1A). Within this clade, a C_3_ group (*M. moricandioides*, *M. foetida*) was sister to the C_3_–C_4_ intermediate species (*M. arvensis*, *M. suffruticosa*, *M. nitens*, *M. sinaica*, *M. spinosa*; Fig. 1A), indicating that the evolution of the C_3_–C_4_ intermediate character is most-parsimoniously explained by a single event in the *Moricandia* genus. Intermediates with smaller, more ellipse-shaped leaves (group I, Fig. 1A; Supplementary Fig. S1) formed a monophyletic group, while the intermediates with mainly longer petioles formed a paraphyletic clade (group II). Lines taxonomically assigned to *M. arvensis* could be found within both groups.

Besides the *Moricandia* C_3_–C_4_ species, very similar features, such as significantly reduced CO_2_ compensation points, occur in *Diplotaxis tenuifolia* and *Brassica gravinae* (Apel *et al.*, 1997; Ueno, 2003, 2011). The development of C_3_–C_4_ intermediacy in these species was clearly independent from the events in *Moricandia* (Fig. 1A). Phylogenetic trees of the Brassicaceae with higher species density (Warwick and Sauder, 2005; Arias *et al.*, 2014) suggest that these species belong to different branches of the tree and that C_3_–C_4_ intermediacy also evolved independently in *D. tenuifolia* and *B. gravinea*. In the list of Apel *et al.* (1997), single measurements of CO_2_ compensation points in *D. muralis* and *D. erucoides* also indicated low CO_2_ compensation points. *Diplotaxis muralis* is a hybrid between *D. viminea* (C_3_) and *D. tenuifolia* (C_3_–C_4_) and usually closer to C_3_ in its gas exchange characteristics (Ueno *et al.*, 2006), but detailed studies are lacking for *D. erucoides*. It is also remarkable that C_3_–C_4_ intermediates were only found in the Oleracea group of the Brassciceae tribe, in the lineage II Brassicaceae (Apel *et al.*, 1997; Warwick and Sauder, 2005; Arias *et al.*, 2014), but no C_3_–C_4_ intermediates have so far been identified in any other subgroup of this large family.

### Physiological features allow differentiation of C_3_ and C_3_–C_4_*Moricandia* lines

Many details of the C_3_–C_4_ intermediate photosynthesis character were investigated in the 1980s and 1990s at the University of Nebraska (see Holaday *et al.*, 1981, 1982; Holaday and Chollet, 1983), in Gatersleben in Germany (see Bauwe and Apel, 1979; Apel *et al.*, 1997), and at the John Innes Centre in Norwich, UK (see Rawsthorne *et al.*, 1988*a*, *b*; Rylott *et al.*, 1998). Since the stock seed material from these initial analyses could no longer be obtained, CO_2_ compensation points in genotyped greenhouse-grown lines were characterized (Fig. 1B) and compared with data from the C_3_ plant *T. hassleriana* and the C_4_ plant *G. gynandra* from the neighbouring Cleomaceae family. The measurements of the CO_2_ compensation points clearly allowed classification of the tested lines as a C_3_, C_3_–C_4_, or C_4_ species (Fig. 1B). All C_3_–C_4_ intermediate lines had CO_2_ compensation points that were significantly lower than in the C_3_ species, but also significantly higher than in the C_4_. On the other hand, no significant differences could be observed among the C_3_–C_4_ intermediate lines.

**Fig. 2.**
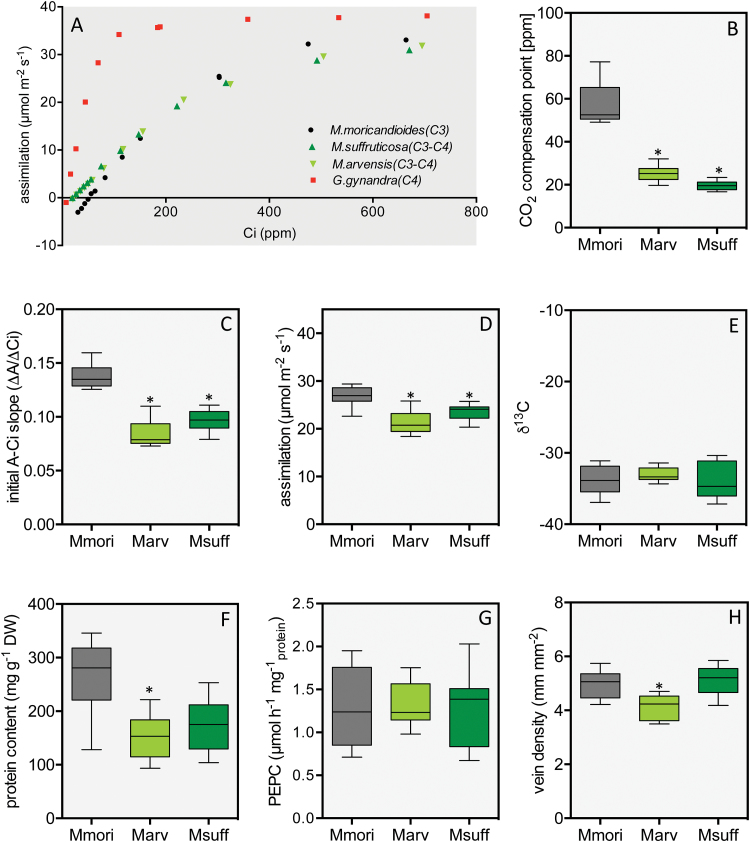
Physiological features in the C_3_ species *M. moricandioides*, and the C_3_–C_4_ intermediate species *M. suffruticosa* and *M. arvensis* line MOR1. (A) Representative examples of *A*-*C*_i_ curves from *M. moricandioides*, *M. suffruticosa*, and *M. arvensis* line MOR1 in comparison with the curve from the C_4_ species *G. gynandra*. (B) CO_2_ compensation points; (C) initial *A*-*C*_i_ slope; (D) assimilation rate under ambient conditions with c_a_ of 400 ppm; (E) δ^13^C signature of leaf material; (F) protein content per plant dry weight; (G) PEPC activity; (H) vein density in the top part of mature leaves. All box-whisker plots show data summarised from 8–10 biological replicates. The asterisks indicate significant differences of the C_3_–C_4_ intermediate species in comparison with the C_3_ species *M. moricandioides*, as determined by a *t*-test (*P≤*0.01). Mmori, *M. moricandioides*; Marv, *M. arvensis* line MOR1; Msuff, *M. suffruticosa*. (This figure is available in colour at *JXB* online.)

The selected accessions *M. moricandioides*, *M. arvensis* MOR1, and *M. suffruticosa* were then grown under controlled environmental conditions in a climate chamber. Under these conditions, the differences in the CO_2_ compensation point of the C_3_ and C_3_–C_4_*Moricandia* species were even more pronounced (Fig. 2A, B). A closer inspection of the shape of *A*-*C*_i_ curves showed that, despite the low CO_2_ compensation point, the curve of the intermediates looked much more similar to the C_3_ curve than the one of the C_4_ species *G. gynandra*, which had a very steep initial Δ*A*/Δ*C*_i_ slope of 0.557 (Fig. 2A). The initial slope of the *A*-*C*_i_ curve is connected to the carboxylation efficiency in the photosynthetic system, the PEPC efficiency in C_4_, and the Rubisco carboxylation efficiency in C_3_ (von Caemmerer, 2000). A comparison of the *A*-*C*_i_ curves in the C_3_ and C_3_–C_4_*Moricandia*s showed that the initial slope was actually steeper in the C_3_ species than in the C_3_–C_4_ intermediate lines (Fig. 2A, C).

The assimilation at ambient CO_2_ was lower in the C_3_–C_4_ intermediate species than in the C_3_ species (Fig. 2D). Differences in conductance were not responsible for this variation, and the maximal CO_2_ assimilation at high CO_2_ reached similar levels in all species tested (see Supplementary Table S4). In addition, measurements of PEPC activity in extracts from mature leaves indicated that PEPC did not play a major role in the primary fixation of CO_2_ in C_3_–C_4_ intermediate *Moricandia* species (Fig. 2G). No significant differences could be observed in the carbon isotope ratio of the C_3_ and C_3_–C_4_ intermediate species (Fig. 2E). The protein content per total dry weight tended to be lower in the intermediates as compared to the related C_3_ species *M. moricandioides*; however, these results were only significant for one C_3_–C_4_ line, *M. arvensis* (Fig. 2F).

### Differences in CO_2_ compensation points are connected to anatomical changes

The development of C_3_–C_4_ intermediate physiology relies on functional specification of metabolism in the MS and BS cells and an increase in the metabolite exchange between the two cell types. Differences among photosynthetic types were therefore expected to be closely connected to changes in the anatomy, which were evaluated on a histological and ultrastructural level.

**Fig. 3.**
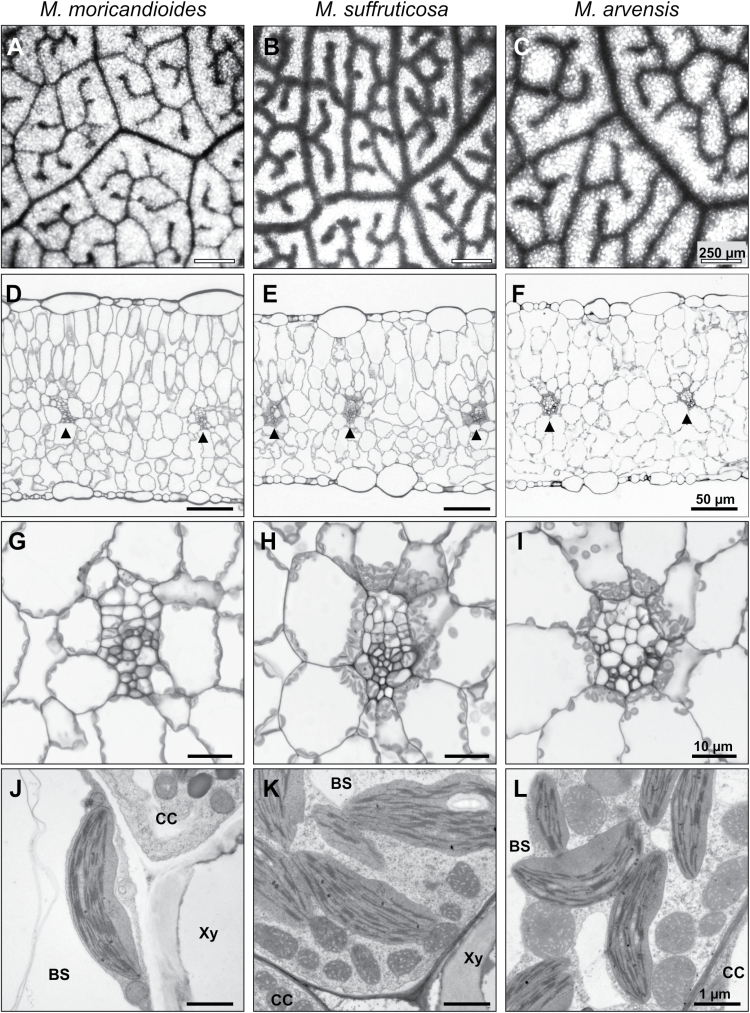
Anatomy of the C_3_ species *M. moricandioides* (A, D, G, J), and the C_3_–C_4_ intermediate species *M.suffruticosa* (B, E, H, K) and *M.arvensis* line MOR1 (C, F, I, L). (A–C) Top view of cleared leaves showing the general vein pattern; (D–F) overview of cross-sections; (G–I) close-up of the arrangement of chloroplasts in the bundle sheath cells; (J–L) transmission electron microscopy of bundle sheath cells with organelles arranged next to the vein cell. Arrow heads indicate vascular bundles. BS, bundle sheath cell; Xy, xylem cell; CC, companion cell.

Measurements of vein length per area revealed that the C_3_–C_4_*Moricandia* species had lower or equal vein density when compared to the C_3_ relative (Fig. 2H). In the top view of cleared leaves, veins of C_3_–C_4_ intermediate species appeared thicker than in the C_3_ species (Fig. 3A–C), probably connected to the large number of chloroplasts, which were centripetally arranged around the veins in the leaf cross-sections (Fig. 3D–I). In-depth ultrastructural analysis showed a high number of mitochondria located predominantly in the cytoplasm between the chloroplasts and the cell wall of adjacent cells of the vascular tissue of C_3_–C_4_ plants (Fig. 3J–L). The C_3_ species *M. moricandioides* had eight cell layers, while *M. suffruticosa* and *M. arvensis* both had one MS cell layer fewer (Supplementary Table S4, Fig. 3D–F). Such a reduction in the MS tissue alone could be responsible for a shift in the leaf cell profile towards a higher proportion of BS cells (McKown and Dengler, 2007).

### Specific metabolite pattern in C_3_–C_4_ intermediates

The metabolite pattern of the leaves is expected to be influenced by species-specific differences as well as by the photosynthesis type. The overall metabolite patterns in *M. moricandioides*, *M. suffruticosa*, and *M. arvensis* were first assessed by principal component analysis (PCA) (see Supplementary Fig. S2A). In the first principal component (PC1), samples from the C_3_ species *M. moricandioides* localised predominantly to the right, next to samples of C_3_–C_4_ intermediate species. PC2 mainly separated the two C_3_–C_4_ intermediate species. The samples from both C_3_–C_4_ intermediates were also characterised by high variation. Three metabolites showed significantly (*P*≤0.01) different concentrations in all tested comparisons (*M. arvensis* vs *M. moricandioides*, *M. suffruticosa* vs M. *moricandioides*, and *M. suffruticosa* vs *M. arvensis*): maleic acid, serine, and threonine (Supplementary Fig. S2B). To distinguish between C_3_ and C_3_–C_4_ intermediate species, we screened for metabolites that significantly differed between the C_3_ and the two C_3_–C_4_ species, but not between the two intermediates. Among the nine metabolites in this category were alanine, glycine, GABA, gluconic acid, leucine, malate, malonic acid, and valine (Supplementary Fig. S2B, Supplementary Table S4).

**Fig. 4.**
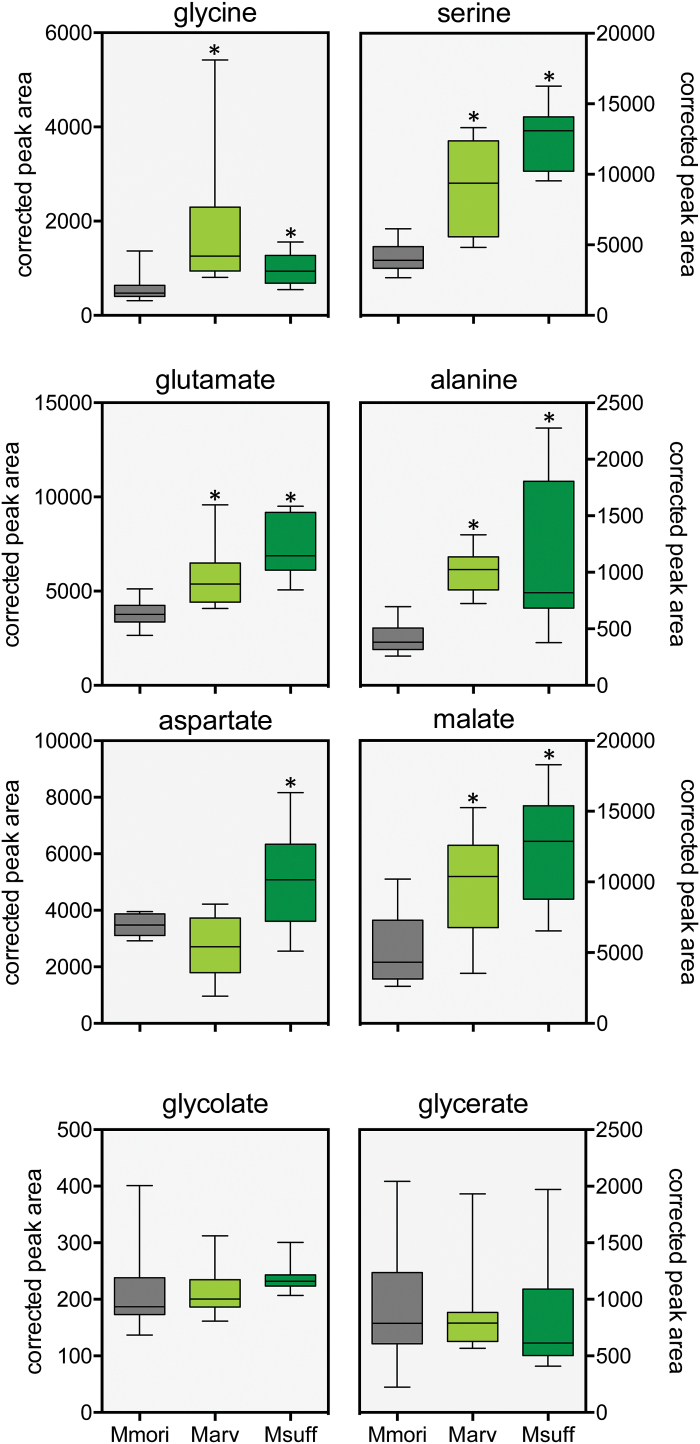
Selected metabolites in the C_3_ species *M. moricandioides*, and the C_3_–C_4_ intermediate species *M. suffruticosa* and *M. arvensis* line MOR1. The box-whisker plots represent summaries of 10–12 biological replicates. The asterisks indicate significant differences between the C_3_–C_4_ and the C_3_ species as determined by a *t*-test (*P*≤0.01). Mmori, *M. moricandioides*; Marv, *M. arvensis* line MOR1; Msuff, *M. suffruticosa*. (This figure is available in colour at *JXB* online.)

The predicted N shuttle metabolites (Mallmann *et al.*, 2014), glutamate, alanine, and malate, had higher concentrations in both intermediate *Moricandia* lines. Aspartate was only increased in *M. suffruticosa* (Fig. 4). Glycolate and glycerate are part of the photorespiratory pathway, but no significant differences in the concentration of these two metabolites were detected in leaves of the C_3_ and C_3_–C_4_*Moricandia* species (Fig. 4), confirming that these metabolites do not play a major role in coordination of metabolism between MS and BS cells.

### Transcript patterns do not show a strong specific C_3_–C_4_ signature

Next-generation sequencing allows an analysis and comparison of the transcriptomes of species for which no reference genome is available by mapping the reads against the minimal transcriptome of *Arabidopsis thaliana* (Brautigam *et al.*, 2011). In all *Moricandia* samples, between 79% and 86% reads were mapped, which is higher than in previous work with Asterales species (Gowik *et al.*, 2011). PCA showed that the transcript pattern was most prominently influenced by the species (see Supplementary Fig. S3A). PC1 clearly separated samples from *M. moricandioides*, *M. arvensis*, and *M. suffruticosa*, while PC2 only separated *M. moricandioides* and *M. arvensis* from *M. suffruticosa* (Supplementary Fig. S3A). Subsequent PCs were already influenced by replicate-specific differences. An influence of the photosynthesis type was not detected in the first three PCs (Supplementary Fig. S3B).

The abundance of 1671 transcripts was significantly different in the C_3_ and the C_3_–C_4_ intermediate leaves, but not between the two intermediates. All of these had changed in both C_3_–C_4_ intermediate species to the same direction: 797 were commonly enhanced in C_3_–C_4_ while 874 were commonly reduced in C_3_–C_4_ (Supplementary Fig. S3C, Supplementary Table S5). GO term analysis of both groups of transcripts revealed quite diverse categories. Only two GO terms were enriched among transcripts enhanced in C_3_–C_4_, while 21 process GO-terms were enriched in transcripts reduced in C_3_–C_4_. The latter included high-level terms such as cellular compound, nitrogen metabolism, and carbohydrate metabolism. The cellular compartment mainly affected by transcript reduction in C_3_–C_4_ intermediates appeared to be the chloroplast (see Supplementary Table S6).

**Fig. 5.**
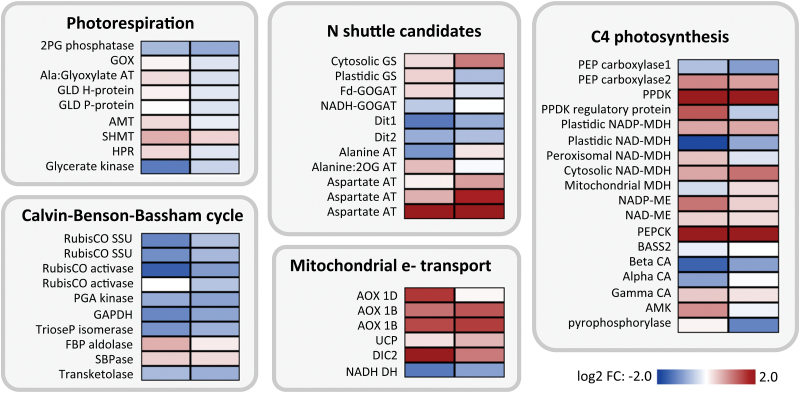
Transcriptional changes in selected pathways. The heat maps indicate the log_2_-fold changes in transcript level between C_3_–C_4_ species *M. arvensis* line MOR1 (left column) and *M. suffruticosa* (right column) and the C_3_ species *M. moricandioides*. Blue indicates reduced transcript abundance in C_3_–C_4_, red indicates enhanced transcript abundance in C_3_–C_4_.

The metabolism in C_3_–C_4_ intermediate leaves is predicted to differ from C_3_ leaves mainly with respect to cellular distribution of photorespiratory processes and subsequent re-adjustment of C and N balance by metabolite shuttle mechanisms (Mallmann *et al.*, 2014). Transcripts predicted to be involved in all these processes were therefore studied in more detail. No changes were observed for transcripts encoding components of the photorespiratory pathway, in particular the GLDP protein, which is only present in the BS cell of C_3_–C_4_ intermediates (Fig. 5). Not all transcripts of the Calvin–Benson–Bassham (CBB) cycle did show significant differences between C_3_ and C_3_–C_4_ species. It was, however, noticeable that almost all transcripts tended to be reduced in the C_3_–C_4_ intermediate species when compared to C_3_ (Fig. 5).

**Fig. 6.**
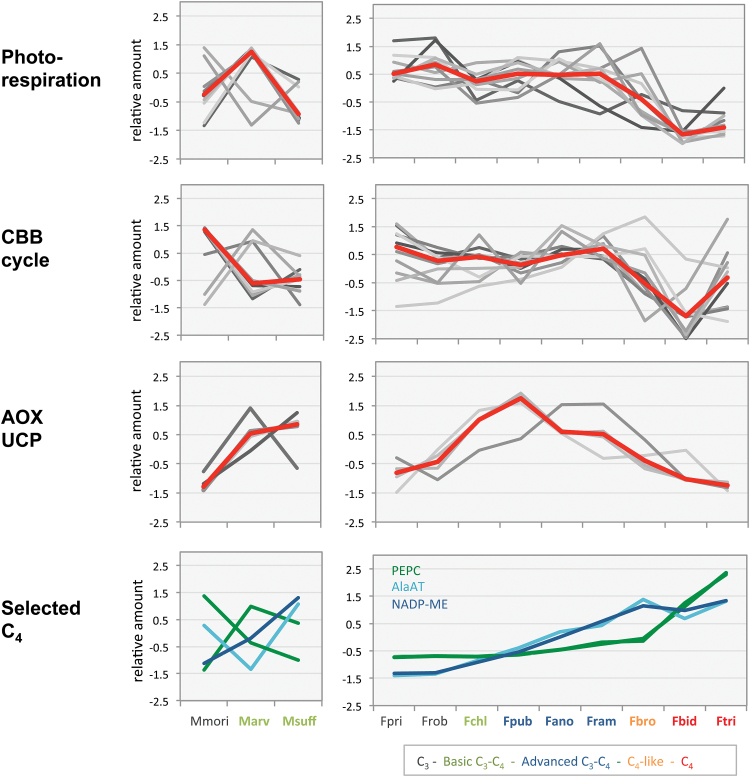
Comparison of the changes in transcript levels of the *Moricandia* species and *Flaveria* species with different degrees of C_4_ features. The graphs show the relative amount (*z*-score normalised data of mean rpm values) from transcripts belonging to the selected pathways. The general trend in the transcription pattern of a pathway is indicated in red as the median of the individual transcripts values. Species abbreviations are: Mmori, *M. moricandioides*; Marv, *M. arvensis*; Msuff, *M. suffruticosa*; Fpri, *F. pringlei*; Frob, *F. robusta*; Fchl, *F. chloraefolia*; Fpub, *F. pubescence*; Fano, *F. anomala*; Fram, *F. ramosissima*; Fbro, *F. brownii*; Fbid, *F. bidentis*; Ftri, *F. trinervia*.

Expression of transcripts belonging to the photorespiratory and CBB pathways is generally lower in C_4_ than C_3_ species (Brautigam *et al.*, 2011). The evolutionary trajectory of pathway expression from C_3_ via C_3_–C_4_ to C_4_ metabolism can be followed in *Flaveria* species and compared to the results from *Moricandia*. No large changes in the average transcript abundance of the photorespiratory pathway were detected in the intermediate *Flaveria* species. The abundance of photorespiratory transcripts decreased only in the C_4_-like *F. brownii* and then even more in the C_4_ species (Fig. 6). The same pattern was also observed for the CBB cycle. A decrease of CBB cycle transcripts in intermediates without increased C_4_ cycle activity, as we found in the two investigated *Moricandia* species, was not observed in *Flaveria* (Fig. 6).

Transcripts encoding enzymes with potential functions in the metabolite shuttles between MS and BS cells were inspected in detail. Only the aspartate aminotransferase (*AspAT*) encoding genes were enhanced in both intermediates compared to the C_3_ species (Fig. 5). Furthermore, transcripts that could potentially be recruited into a C_4_ cycle were tested for their pattern (Fig. 5). In *Flaveria*, basic C_3_–C_4_ intermediates were characterised by increases in alanine aminotransferase (*AlaAT*) and NADP-malic enzyme (*NADP-ME*) transcripts, in the evolutionary series this was followed by increases in *PEPC* transcripts, which were enhanced in all but one of the basic *Flaveria* intermediates (*F. chloraefolia*; Fig. 6). In C_3_–C_4_ intermediate *Moricandia* species, *AlaAT* transcripts were not enhanced, and only slight increases in *NADP-ME* transcripts were observed (Fig. 5). Another potential C_4_ decarboxylating enzyme, *PEPCK*, showed enhanced transcript abundance in the C_3_–C_4_*Moricandia* intermediates (Fig. 5; Supplementary Table S7). The same was true for the *PPDK* transcripts. Two *PEPC* transcripts with C_3_-like characteristics (Paulus *et al.*, 2013) were found in noticeable amounts in *Moricandia* leaves. The higher-abundant form was actually reduced compared to the C_3_ transcriptome, and only the lower-abundant form was enhanced in the intermediates (Fig. 5; Supplementary Table S7).

The GLD/SHMT reaction in the BS also produces NADH and this may require adjustments of the redox balance in the cells. Strong increases were observed for transcripts encoding alternative oxidases (*AOX*) in the C_3_–C_4_ intermediates. The same tendency was found for the uncoupling protein (*UCP*) and the dicarboxylate transporter *DIC2* (see Supplementary Table S7). A NADH dehydrogenase transcript on the other hand was reduced in the C_3_–C_4_ leaves (Fig. 5). Increases in *AOX* and *UCP* transcripts were not unique to *Moricandia*, but were also observed in the C_3_–C_4_*Flaveria* intermediates. In the C_4_-like and C_4_*Flaveria* species, the *AOX* and *UCP* transcripts decreased again compared to the C_3_ plant. Hence the increase in *AOX* transcript abundance was a common feature in C_3_–C_4_ intermediate *Moricandia* and *Flaveria* species (Figs 5 and 6).

### *Moricandia* species possess only a single copy of the *GLDP* gene

**Fig. 7.**
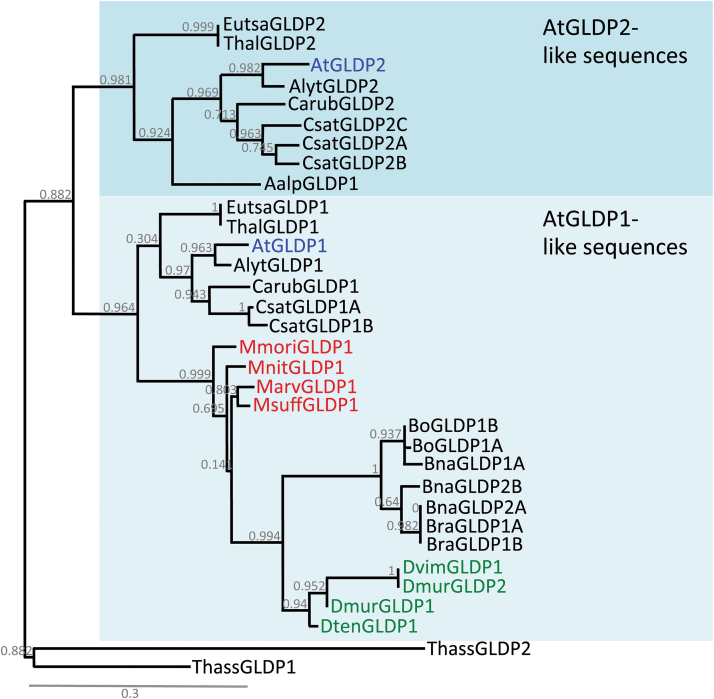
Phylogenetic tree of the glycine decarboxylase P-protein (GLDP) coding sequences in selected Brassicaceae. The *GLDP* copies from *Arabidopsis thaliana* are highlighted in blue, *Moricandia* species are in red, and *Diplotaxis* species are in green. Species abbreviations are: At, *Arabidopsis thaliana*; Alyt, *Arabidopsis lyrata*; Aalp, *Arabis alpina*; Bna, *Brassica napus*; Bo, *B. oleraceae*; Bra, *B. rapa*; Eutsa, *Eutrema salsugineum*; Thal, *Thellungiella halophile*; Csat, *Camelina sativa*; Carub, *Capsella rubella*; Thass, *Tarenaya hassleriana*; Mmori, *Moricandia moricandioides*; Mnit, *Moricandia nitens*; Marv, *Moricandia arvansis* line MOR1; Msuff, *Moricandia suffruticosa*; Dvim, *Diplotaxis viminea*; Dten, *Diplotaxis tenuifolia*; Dmur, *Diplotaxis muralis*). Branch support is determined by an approximate likelihood ratio test.

The number of *GLDP* copies and their phylogenetic relationship was investigated in the Brassicaceae. Only species where full genome information was available were considered for the comparison. *Arabidopsis thaliana* has two copies of the gene, *AtGLDP1* and *AtGLDP2* (Engel *et al.*, 2007). Comparison with other Brassicaceae revealed that other species from the Brassicaceae lineage I (*Arabidopsis lyrata*, *Camalina sativa*, *Capsella rubella*) as well as species from the extended lineage II (*Eutrema salsugineum*, *Thellungiella halophila*) also possess transcripts with high similarity to both *Arabidopsis* genes (Fig. 7). From *Arabis alpina* only the *AtGLDP2*-like gene was identified. In the *Brassica* species *B. rapa*, *B. oleracea*, and *B. napus* on the other hand, only *AtGLDP1*-like copies were found (Fig. 7). Transcriptomes from mature leaves of *M. moricandioides*, *M. suffruticosa*, *M. arvensis*, *Dipoltaxis tenuifolia*, *D. viminea*, and *D. muralis* also assembled only copies with high similarity to the *AtGLDP1* gene. In all these species, *GLDP* was represented by one unique transcript. Two transcripts were assembled only in *D. muralis*, one with a high similarity to the sequence of *D. tenuifolia* and the other one with high similarity to the *D. viminea* sequence. Since *D. muralis* is a hybrid between these two species, this was expected and underlines the successful assembly of the *GLDP* gene sequences in our study. An assembly of gene sequences from a leaf transcriptome could still miss copies that are simply not expressed at all in the leaves examined. The complete absence of *AtGLDP2*-like sequences also in the genome of the sequenced *Brassica* species, however, indicated that the *AtGLDP2* copy was absent in the whole Brassiceae subgroup containing *Brassica*, *Moricandia*, and *Diplotaxis* species, most likely by loss at the base of Brassiceae subgroup.

## Discussion

### Evolution from C_3_ to C_3_–C_4_ was promoted by lack of a second *GLDP* copy in the Brassiceae

Evolution of C_4_ photosynthesis is not equally distributed across the plant kingdom, being frequent in some groups but completely absent from many others with similar growth forms (Sage *et al.*, 2011*a*; Sage, 2016). In the Brassicaceae we find no true C_4_, but possibly three independent C_3_–C_4_ lines (*Moricandia*, *Diplotaxis tenuifolia*, *Brassica gravinae*), and all these lines belong to the Oleracea group of the Brassiceae (Warwick and Sauder, 2005; Arias *et al.*, 2014).

The decisive step from C_3_ to C_3_–C_4_ intermediacy is associated with a shift of the activity of photorespiratory GLD from ubiquitous expression to almost exclusive expression in the BS (Heckmann *et al.*, 2013; Khoshravesh *et al.*, 2016). Detailed studies of the promoter sequences of the GLDP in *Flaveria* have shown that the regulatory elements mediating BS-specific expression were already present in the C_3_ ancestors (Schulze *et al.*, 2013). *Flaveria* species possess two copies of the *GLDP* gene: one is ubiquitously expressed in the leaf tissue (*GLDPB*), while transcripts of the other one were found exclusively in the BS (*GLDPA*). The transition from C_3_ to C_3_–C_4_ photosynthesis in *Flaveria* was then realised via a gradual decrease and finally pseudogenization of the ubiquitously expressed copy and exclusive expression of the BS-specific gene (Schulze *et al.*, 2013).

*Arabidopsis thaliana* belongs to the lineage I of the Brassicaceae and also possesses two copies of the *GLDP* gene, *AtGLDP1* and *AtGLDP2*, and both are abundantly expressed in leaf tissue (Engel *et al.*, 2007). Orthologs of the genes were also detected in species from the same lineage and also in the extended lineage II of Brassicaceae. Only in the Brassiceae subgroup, which includes all known C_3_–C_4_ Brassicaceae species, was the *AtGLDP2*-like copy missing (Fig. 7). The step from C_3_ to C_3_–C_4_ photosynthesis in the Brassicaceae was apparently facilitated by loss of the *GLDP2* copy. Analysis of the promoter region from the *AtGLDP1* gene revealed the presence of an MS (M-box) as well as a BS/vein (V-box) -specific element and these were highly conserved throughout the Brassicaceae family (Adwy *et al.*, 2015). Changes in the M-box of the promoter could therefore easily lead to loss of gene function in the mesophyll, and without a second copy of the gene, BS-specific *GLDP* expression typical for the C_3_–C_4_ species could be realised, driven by the V-box. This scenario is supported by the absence of the M-box but the conserved presence of the V-box in the *GLDP* promoter of the C_3_–C_4_ species *Moricandia nitens* (Zhang *et al.*, 2004; Adwy *et al.*, 2015). In *Flaveria*, it has been hypothesised that a gradual decrease of MS *GLDP* expression might have been crucial for the adjustment of intercellular metabolism (Schulze *et al.*, 2013). It will be interesting to investigate whether single nucleotide changes are sufficient to completely disrupt the function of the Brassicaceae M-box. So despite the differences in the order of events in *Flaveria* and *Moricandia*, in both genera BS-specific elements were present in the *GLDP* promoter sequences of C_3_ ancestors and the transition from C_3_ to C_3_–C_4_ photosynthesis could be promoted by small changes in the genome.

### C_3_–C_4_ characteristics are stable in different *Moricandia* species

*Moricandia* species had been characterised for their specific physiological, anatomical, and biochemical properties (Bauwe and Apel, 1979; Rawsthorne *et al.*, 1988*a*, *b*; Brown and Hattersley, 1989), but direct comparisons of the results from the different laboratories could be problematic because the features investigated might vary among different accessions of the same species (Sayre and Kennedy, 1977). We therefore tested CO_2_ compensation points and phylogenetic relationships between one C_3_*M. moricandioides* line and eight different C_3_–C_4_ intermediate lines. In the phylogenetic tree, all C_3_–C_4_ intermediates of *Moricandia* formed a monophyletic clade, indicating that the evolution of C_3_–C_4_ photosynthesis probably occurred once, with subsequent speciation (Fig. 1A). The CO_2_ compensation points of all the C_3_–C_4_ lines tested were significantly lower than in C_3_ relatives and higher than in the C_4_ species *G. gynandra*, but the lines did not differ amongst each other (Fig. 1B).

In the C_3_–C_4_ accessions *M. arvensis* line MOR1 and *M. suffruticosa*, the reduction in CO_2_ compensation point was closely associated with an increase in organelle number and their centripetal arrangement in the BS cells of the mature leaf (Fig. 3). A very similar picture has been described for the C_3_–C_4_ species *Moricandia spinosa* (Brown and Hattersley, 1989) and *M. arvensis* (Holaday *et al.*, 1981), as well as C_3_–C_4_ species from other plant families (McKown and Dengler, 2007; Muhaidat *et al.*, 2011; Khoshravesh *et al.*, 2016). BS cells in the C_3_–C_4_ intermediates are still in direct contact with the intercellular space and CO_2_ can diffuse in and out of the cell. Therefore, the efficiency of the glycine shuttle and re-fixation of the released CO_2_ depends on the close arrangement of the mitochondria, where CO_2_ is released, and the chloroplasts, where the carboxylation reaction of Rubisco can profit from the proximate increase in CO_2_ concentration.

After establishment of the photorespiratory glycine shuttle, further fitness gains are predicted by support of the glycine shuttle by C_4_ acids, which serve as carbon backbones for re-assimilation of photorespiratory ammonia (Heckmann *et al.*, 2013; Mallmann *et al.*, 2014). The majority of *Flaveria* C_3_–C_4_ intermediates are characterised by enhanced PEPC activity and a limited C_4_ cycle (Vogan and Sage, 2011; Mallmann *et al.*, 2014). In C_3_–C_4_ intermediate *Moricandia* species, the PEPC activity was not changed compared to the C_3_ species (Fig. 2G). Earlier measurements of PEPC activity in *M. arvensis* showed a slight two-fold increase in comparison to the C_3_ species *M. foetida* (Holaday *et al.*, 1981). However, ^14^C labelling experiments gave no further evidence for a significant contribution of C_4_ acids to the photosynthetic carbon assimilation (Holaday and Chollet, 1983; Hunt *et al.*, 1987). Values for δ^13^C, which would indicate a substantial contribution of PEPC to primary CO_2_ fixation, were also indistinguishable between C_3_ and C_3_–C_4_*Moricandia* species. In *Flaveria*, the installation of the glycine shuttle led to implementation of different degrees of the C_4_ cycle, including true C_4_ photosynthesis, but in the *Moricandia* species analysed similar developments were not accompanied by a substantial C_4_ pathway contribution.

### C_3_–C_4_ specific metabolism influences metabolite but not transcript patterns in *Moricandia*

**Fig. 8.**
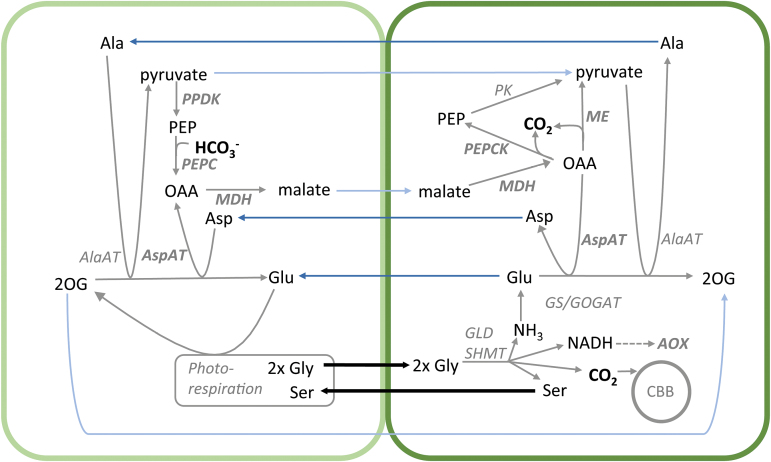
Model of metabolite shuttle network active between the mesophyll cells (left) and bundle sheath cells (right) of C_3_–C_4_ intermediate *Moricandia* species. The inactivity of the photorespiratory glycine decarboxylating complex in the mesophyll cells leads to glycine accumulation and transport to the bundle sheath. In the mitochondria of bundle sheath cells two molecules of glycine are converted by the GLD/SHMT complex to serine, CO_2_, NH_3_, and NADH. In the adjacent chloroplasts the bundle sheath Rubisco is exposed to enhanced CO_2_ conditions. The imbalance created by parallel release of NH_3_ requires further adjustment of C and N metabolism that is probably realised by a whole network of reactions, including additional shuttling of amino acids from the bundle sheath to the mesophyll (dark blue arrows), and re-shuttling of organic acids (light blue arrows). Enzymes highlighted in bold could be associated with an increased abundance of at least one transcript copy.

The absence of GLD is thought to induce enhanced glycine concentration in the MS, followed by diffusion of the metabolite to the BS (Rawsthorne and Hylton, 1991). As expected, an increase in the glycine concentration was detectable in total leaf extracts from *M. arvensis* and *M. suffruticosa* (Fig. 4). Serine, as the direct product of the GLD/SHMT, reaction is most likely one of the metabolites transported back to the MS cell. Just like glycine, its steady-state pool is typically increased in C_3_–C_4_ intermediate leaf extracts and it is characterised by a high turnover rate in the illuminated leaf (Fig. 4; Rawsthorne and Hylton, 1991). The pools of metabolites suggested to maintain N-balance (e.g. glutamate, alanine, malate) were also increased in the C_3_–C_4_*Moricandia* lines when compared to the C_3_ relative species; only aspartate was enhanced in just one intermediate line. A strong preference for one of the suggested shuttle mechanisms could, however, not be detected. The results tend to suggest that many shuttles contribute to the metabolic balancing between the cells (Fig. 8), and it is very well possible that further metabolites are involved.

In contrast to the metabolite patterns, a C_3_–C_4_-related transcript pattern could not be detected in the PCA of *Moricandia* transcriptomes. The partly random enrichment of GO-terms in the commonly up- and down-regulated transcripts also suggested that species-specific differences had a major influence on the transcript pattern. The results differed considerably from the picture obtained for comparisons of transcriptomes from C_3_ and C_4_ species (Brautigam *et al.*, 2011, 2014; Gowik *et al.*, 2011), which were always characterised by a very strong C_4_ signature with very high abundance of all transcripts encoding the C_4_ photosynthesis proteins, including *PEPC*, *PPDK*, *ME*, *NADP-MDH*, *AspAT*, and adenosine monophosphate kinase (Brautigam *et al.*, 2014). Changes in the *Moricandia* lines were on a much smaller scale, but some transcripts encoding enzymes associated with C_4_ metabolism, such as *PPDK*, *PEPCK*, a plastidic *NADP-MDH*, a cytosolic *NAD-MDH*, and three copies of *AspAT*, were also enhanced in both C_3_–C_4_ intermediate species. The same was true for one *PEPC* copy (Supplementary Table S5).

In *Flaveria*, only *F. chorifoliae* displayed a similar level of C_3_–C_4_ metabolism as the *Moricandia* species tested, with no significant contribution of PEPC and the C_4_ cycle. However, even this basic C_3_–C_4_ intermediate species showed increases in transcript abundance of *AlaAT* and *NADP-ME*, and these changes were associated with the operation of the N-balancing shuttle mechanisms. The fact that the transcript changes in the C_3_–C_4_ intermediate *Moricandia*s were usually moderate or small compared with true C_4_ species supported the hypothesis that there was not one main shuttle operating. Not all steps predicted in the model shown in Fig. 8 were accompanied by increases in transcript levels. This might not be necessary because the required enzymes are not only present in a C_3_ background (Aubry *et al.*, 2011), but also of high enough abundance. It is furthermore possible that transcripts did not change in their general abundance between C_3_ and C_3_–C_4_ species, but that they were affected in their post-transcriptional regulation or cellular distribution instead. Overall, metabolite as well as transcript patterns indicated that the N metabolism between MS and BS was adjusted by multiple pathways.

### Redox balance requires transcriptional changes

When the GLD reaction is shifted to the BS, it increases not only the release of CO_2_ and NH_4_ in the BS mitochondria, but also produces high amounts of NADH (Fig. 8). In *Moricandia*, three *AOX* and one *UCP* transcripts were more abundant in the leaves of C_3_–C_4_ intermediate species than in the C_3_ relative, suggesting that re-balancing the redox metabolism of the mitochondria is supported by alternative electron transport. Increases in *AOX* are usually found under stress, but *AOX* expression is also affected in photorespiratory mutants (Voss *et al.*, 2013). The association of enhanced alternative electron transport in C_3_–C_4_ photosynthesis could be verified by comparisons with the transcript patterns in different *Flaveria* species. Increases in *AOX* transcripts were also present in the intermediate species but they returned to C_3_ levels in the C_4_ species (Fig. 6), indicating that redox balance is harmonised again after full implementation of the C_4_ cycle. This model predicts the irretrievable loss of the mitochondrial NADH transported by the photorespiratory pump.

### Anatomical and environmental constrains could be responsible for impeded evolution towards C_4_ in *Moricandia*

In the model presented by Mallmann *et al.* (2014), the initial shift of the GLD in the C_3_–C_4_ intermediates promotes a smooth transition to C_4_ by gradual enhancement of the C_4_ cycle, but it does not provide a straightforward explanation why some species remain stuck in intermediacy. In *Moricandia*, the analysis of potential C_4_ cycle genes indicated that they are expressed, albeit at low level, in the intermediates and are theoretically capable of forming a C_4_ cycle. So possible reasons for abidance of *Moricandia* in the C_3_–C_4_ state could be lack of time or the absence of some genetic, anatomic, or environmental drivers for the transition to C_4_ to take place (Mallmann *et al.*, 2014; Heckmann, 2016).

Estimates of the time of split between C_3_ and C_3_–C_4_*Moricandia*s are between 11 Ma (Fig. 1A) and 2 Ma (Arias *et al.*, 2014). The same period was predicted to have passed since the separation of C_3_ and C_3_–C_4_ intermediate *Diplotaxis* species (Fig. 1A). In *Flaveria*, one of the youngest lines evolving C_4_ photosynthesis, C_3_–C_4_ metabolism is thought to have evolved about 3 Ma ago and, in at least one line, evolution to full C_4_ photosynthesis was completed about 1–0.5 Ma ago (Christin *et al.*, 2011*a*). Although accurate timing of these evolutionary events is difficult, the results indicate that evolution from C_3_–C_4_ to C_4_ might have been generally possible in the 2–11 Ma that elapsed since the origin of C_3_–C_4_ in *Moricandia*, but it would probably depend on several beneficial pre-conditions. Stability of C_3_–C_4_ metabolism for several Ma has also been described for a second lineage in *Flaveria* (*F. sonorensis*; Christin *et al.*, 2011*a*) and *Mollugo* (Christin *et al.*, 2011*b*).

Environmental conditions promoting C_4_ evolution can generally be associated with conditions of high photorespiration, such as high temperatures and C limitation of photosynthesis, which can be found in hot, open environments with water limitation and high salinity (Osborne and Sack, 2012; Brautigam and Gowik, 2016). In many habitats, nutrients other than carbon, for example bio-available nitrogen or phosphorus, restrict plant growth (Korner, 2015). The low carboxylation efficiency of *Moricandia* intermediates, as indicated by low initial slopes in the *A*-*C*_i_ curves (Fig. 2C), point to low N-content in leaves (Sage *et al.*, 1987), probably due to reduced levels of Rubisco and CBB cycle enzymes (Fig. 5). Lower leaf N-content was supported by higher C/N ratios and lower protein content per leaf dry-weight (Fig. 2). The advantages of intermediate *Moricandia*s were thus probably limited to very low CO_2_ partial pressures, as occur when stomata are closed due to water limitations, while C_3_*Moricandia*s reached higher assimilation rates under ambient CO_2_, as encountered when stomata are open. The low leaf protein content pointed to an evolutionary history of adaptation to N-limited environments. A comparison with *A*-*C*_i_ curves from other C_3_–C_4_ intermediate species showed that the phenotype is specific for *Moricandia*. In intermediate species of *Heliotropium*, the carboxylation efficiency of C_3_–C_4_ intermediates was slightly higher than in related C_3_ species (Vogan and Sage, 2011), and the carboxylation efficiency presented for C_3_–C_4_ intermediate *Flaveria* species are also similar to the C_3_ relatives (Dai *et al.*, 1996). Expression of CBB cycle genes was only reduced in *Flaveria* species with at least C_4_-like metabolism, while transcription remained comparable to C_3_ species in all C_3_–C_4_ intermediates (Fig. 6; Mallmann *et al.*, 2014). In both the C_3_–C_4_ intermediate *Moricandia* lines that were tested, on the other hand, the CBB cycle genes were already reduced at the basic intermediate state (Figs 5 and 6). Transcripts belonging to nitrogen as well as carbohydrate metabolism are enriched in the group of genes commonly reduced in the intermediate species. Thus, possibly both C and N limitation promoted the evolution of C_3_–C_4_ intermediacy in these species.

Finally, *Moricandia* provided new insights into the importance of anatomic enablers not only for the transition from C_3_ to C_3_–C_4_ but also for further evolution towards C_4_. Activation of BS cells and high vein density are essential pre-conditions for establishment of an efficient C_4_ cycle (Christin *et al.*, 2013; Khoshravesh *et al.*, 2016). The efficiency of the glycine shuttle and connected C- and N-balancing mechanisms depend on enhanced metabolite exchange between the MS and BS cells and are therefore also dependent on a limited distance between the two cell types. The importance of the narrow vein spacing increases with the increasing contribution of a C_4_ cycle in advanced C_3_–C_4_ intermediates and finally through to full C_4_ (McKown and Dengler, 2007). Plant families in which C_4_ photosynthesis evolved such as *Flaveria* and *Heliotropium* are generally characterised by vein densities considerably higher than in *Moricandia* (McKown *et al.*, 2005; Muhaidat *et al.*, 2011). It is therefore possible that limited anatomical pre-conditions hampered evolution to C_4_ in the Brassiceae.

## Conclusions

Current models suggest that after implementation of the photorespiratory CO_2_ pump, re-balancing of N and C metabolism promotes further shuttle mechanisms involving C_4_ metabolites between the MS and BS cells, and finally installation of highly efficient C_4_ photosynthesis (Mallmann *et al.*, 2014; Brautigam and Gowik, 2016). In *Moricandia*, the installation of a glycine shuttle was definitely successful, and they possessed BS-specific GLDP expression, low CO_2_ compensation points, and BS cells with a high number of centripetally arranged organelles. The metabolite pattern also suggests the activity of additional metabolite shuttles in the intermediates leaves. Establishment of the C_4_ cycle was apparently not hampered as the C_4_ cycle genes were present and expressed. Thus far, the situation in *Moricandia* does not look very different from *Flaveria*, but while some *Flaveria* lines progressed to C_4_, all *Moricandia* lines remained at the basic intermediate state. Lack of progression to C_4_ in the Brassicaceae could still be connected to chance, but our *Moricandia* data now provide evidence for possible constrains on the path to C_4_, namely anatomical limitation of efficient metabolite exchange or insufficient evolutionary pressure due to limitation in nutrients other than carbon, i.e. nitrogen. In contrast to C_3_–C_4_ lines with C_4_ relatives, intermediate Brassicaeae grow in colder climates (MR Lundgren and PA Christin, unpublished data), so pressure to reduce photorespiration might also be limited. In the end, limited environmental pressure and anatomical constrains might have led to metabolic balancing by multiple pathways rather that continued promotion of the C_4_ cycle in *Moricandia*. The analyses of additional intermediates with no closely related C_4_ species, especially with respect to their leaf architecture and N metabolism, will hopefully provide further glimpses into the evolution of intermediacy and of C_4_.

## Supplementary data

Supplementary data are available at *JXB* online.

Figure S1. Phenotypes of the tested *Moricandia* lines.

Figure S2. Statistical summary of *Moricandia* metabolite patterns.

Figure S3. Statistical summary of *Moricandia* transcript patterns.

Table S1. ITS sequences extracted from the NCBI database.

Table S2. Sequences for the glycine decarboxylase P-protein.

Table S3.: Protocol for combined conventional and microwave-proceeded fixation, dehydration, and resin embedding of *Moricandia* leaf sections for histological and ultrastructural analysis.

Table S4. Summary of metabolite, element, gas exchange, anatomy, and protein measurements.

Table S5. Transcripts significantly different in abundance between the three *Moricandia* species (*M. moricandioides, M. arvensis* line MOR1 and *M. suffruticosa*)

Table S6. GO-terms enriched in commonly regulated transcripts in the comparison between C_3_–C_4_ and C_3_*Moricandi*a species, but not different between the two C_3_–C_4_*Moricandia* species.

Table S7. Changes in abundance of transcripts belonging to selected pathways.

## Supplementary Material

Supplementary_Tables_S1_S3_Figures_S1_S3

Supplementary_Tables_S4_S7
