# Supplementary material for: Photosynthesis in C3–C4 intermediate Moricandia species
Source: J Exp Bot. 2016 Oct 19;68(2):191–206. doi: 10.1093/jxb/erw391 (PMC5853546; doi:10.1093/jxb/erw391)
Supplement: Supplementary_Tables_S1_S3_Figures_S1_S3 [file erw391_suppl_supplementary_tables_s1_s3_figures_s1_s3.pdf]

Supplementary table S1. ITS sequences extracted from the NCBI database

Moricandia spinosa	EF601907.1
M. sinaica	EF601906.1
M. foetida	EF601902.1
M. foleyi	EF601903.1
Sinapis alba	AY662291.1
Diplotaxis harra	AY722449.1
D. catholica	AY722446.1
D. erucoides	AY722448.1
Brassica gravinae	AY722419.1
B. napus	DQ003665.1
B. nigra	EF601911.1
B. juncaceae	DQ003680.1
Raphanus sativus	AY662290.1
Gynandropsis gynandra	HM044254.1
Tarenaya hassleriana	AY662284.1
A. thaliana	AJ232900.1

Supplementary table S2. Sequences for glycine decarboxylase P-protein

Arabidopsis thaliana	At4g33010, At2g26080
A. lyrata	XM_002867166, XM_002880695
Arabis alpina	KFK32542
Brassica napus	GSBRNA2T00130674001, GSBRNA2T00121192001, XM_013803942
B. oleraceae	Bol013540, XM_013739860
B. rapa	XM_009126871, Bra011405
Eutrema salsugineum	XM_006408783, XM_006412338
Thellungiella halophile	AK353107; AK352541
Camelina sativa	XM_010434326, XM_010439516, XM_010419085, XM_010474335, XM_010431321
Capsella rubella	XM_006282992, XM_006296411
Tarenaya hassleriana	XM_010527760; XM_010521549
Moricandia nitens	AY544772

Supplemental Table S3: Protocol for combined conventional and microwave-proceeded fixation, dehydration and resin embedding of *Moricandia* leaf sections for histological and ultrastructural analysis.

Combined conventional and micowave processing				
**Process**	**Reagent**	**Power [W]**	**Time [sec]**	**Vacuum [mm Hg]**
**1. Primary fixation**	2.0% (v/v) glutaraldehyde and 2.0% (v/v) paraformaldehyde in 0.05 M cacodylate buffer (pH 7.3)	150	60	0
		0	60	0
		150	60	0
		0	60	0
		150	60	0
		additional incubation for 15 minutes on a shaker at RT		
**2. Wash**	1x 0.05 M cacodylate buffer (pH 7.3) And 2x aqua dest.	150	45	0
**3. Secondary fixation**	1% (v/v) osmiumtetroxide in aqua dest.	0	60	10
		80	120	10
		0	60	10
		80	120	10
		additional incubation for 15 minutes on a shaker at RT		
**4. Wash**	3x aqua dest.	150	45	0
**5. Dehydration**	acetone series:30%, 40%, 50%, 60%, 70%, 80%, 90%, 1x 100%.	150	45	0
	propylenoxide	15 minutes on a shaker		
**6. Resin infiltration**	Spurr´s resin in propylenoxide: 20%, 40%, 60%, 80% and 100% Spurr	250	180	5
	100% Spurr	12 hrs on shaker for.at RT		
**7. Polymerisation**	24 hrs at 70°C in a heating cabinet.			

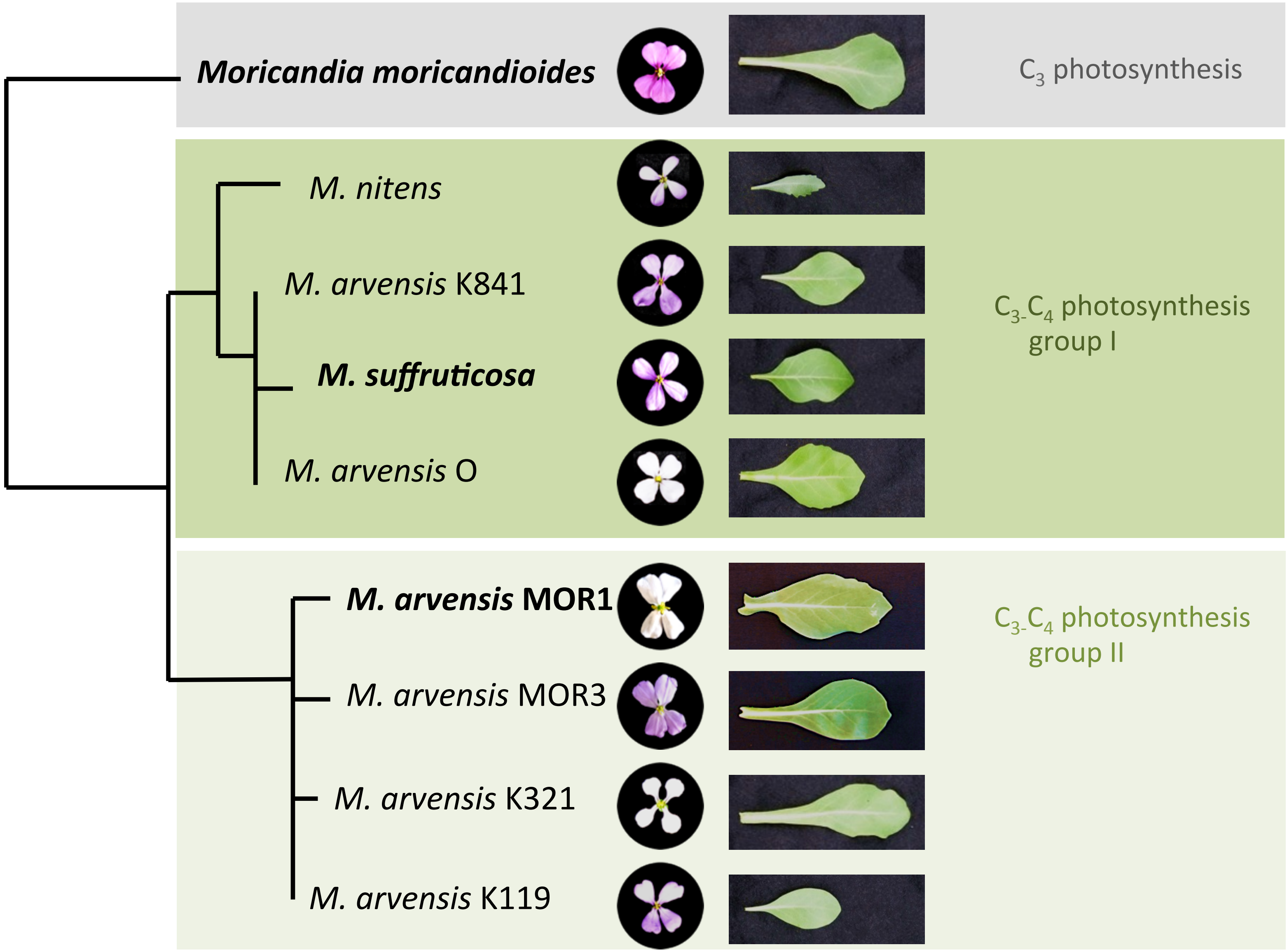

Supplemental Figure S1: Phenotype of tested Moricandia lines
(*Moricandia moricandioides*: 04-0393-10-00 from Botanic Gardens Osnabrück; *M. arvensis*: line 12-0020-10-00 from Botanic Gardens Osnabrück, lines 0119708, 0000321, 0084187 from Royal Botanic Gardens in Kew, lines MOR1 and MOR3 from IPK Gatersleben; *M. suffruticosa*: line 0105433 from Royal Botanic Gardens in Kew; *M.nitens*: 0209858 from Royal Botanic Gardens in Kew)

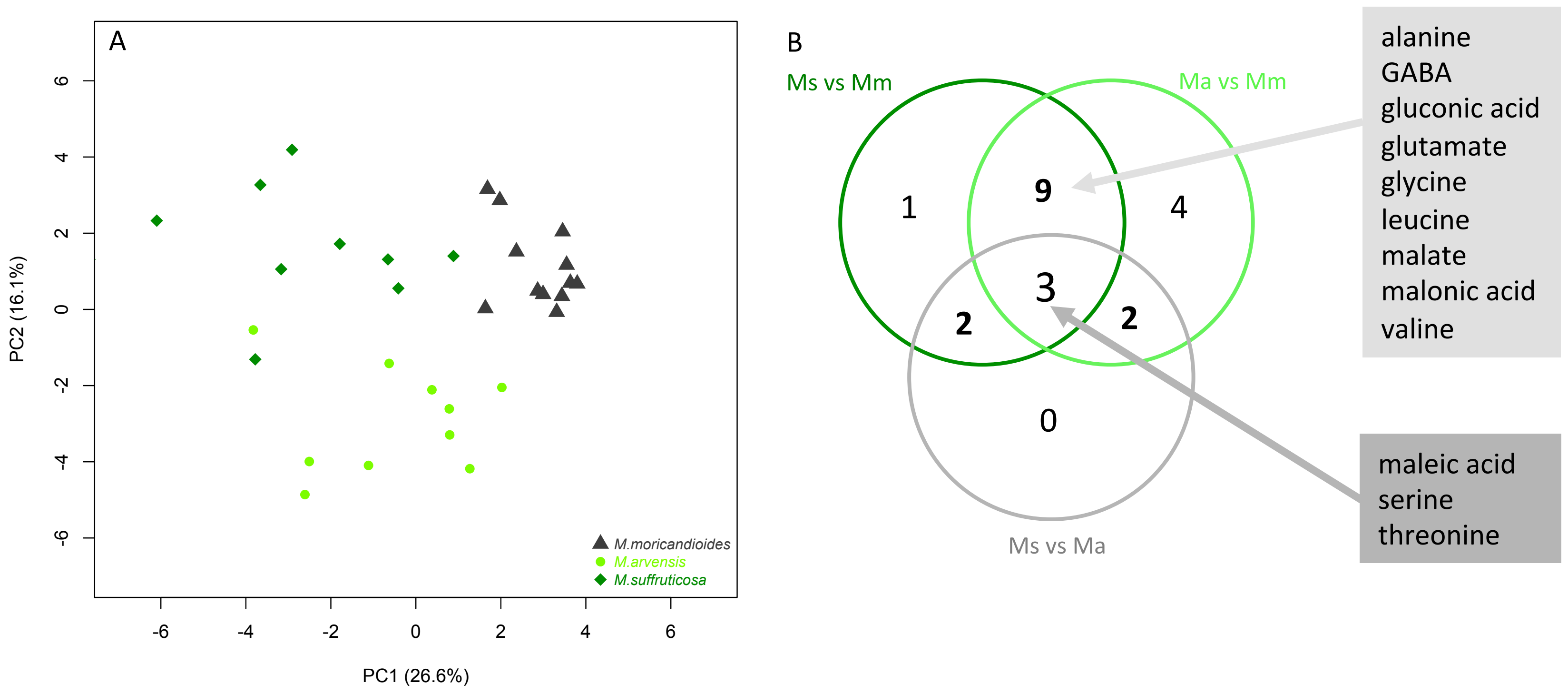

Supplemental Figure S2. Statistical summary of *Moricandia* metabolite patterns
A. Principal component analysis of z-score normalised metabolite data; B. Venn diagram showing intersection of metabolites with significant concentration differences (t-test, $p \leq 0.01$) between the three *Moricandia* species (Mm= *M. moricandioides*; Ma= *M.arvesnis* line MOR1; Ms= *M.suffruticosa*)

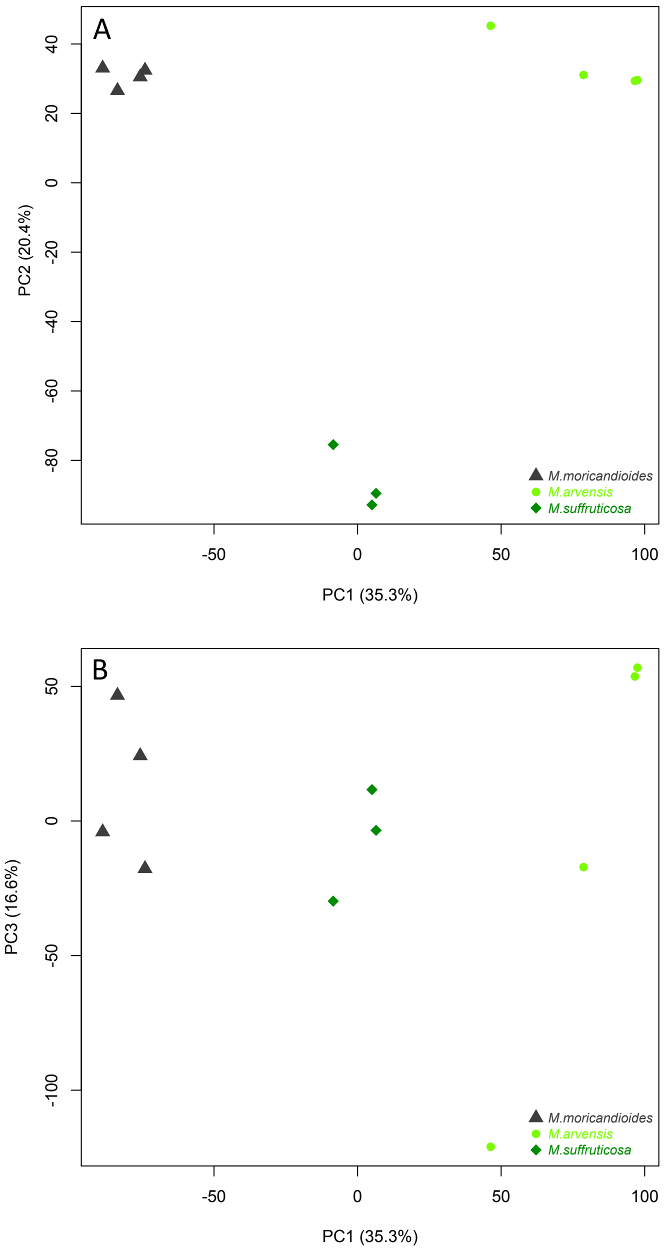

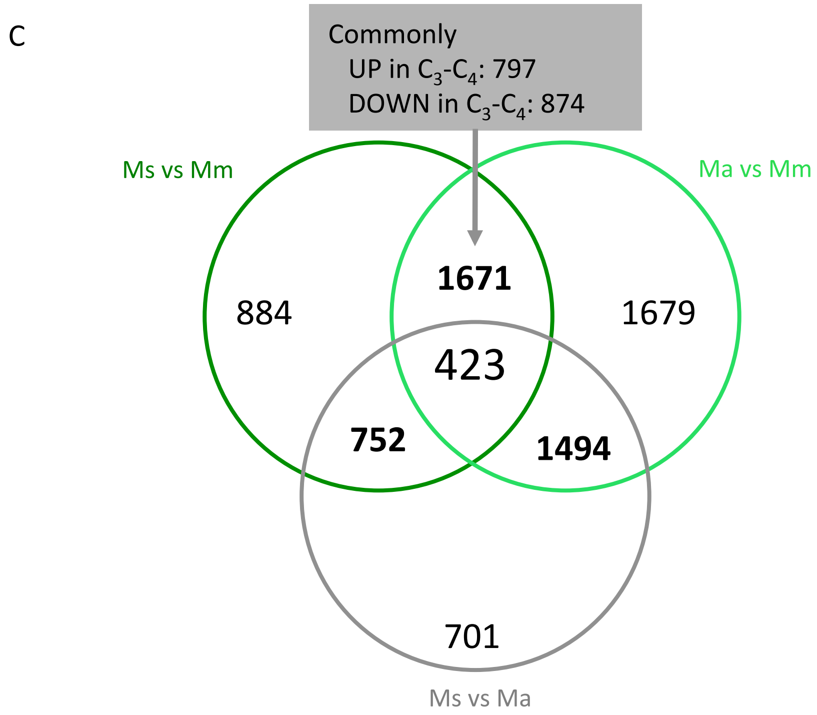

Supplemental Figure S3. Statistical summary of *Moricandia* transcript patterns

A and B. Principal component analysis of log2 normalised transcripts data; C. Venn diagram showing intersection of transcripts with significant concentration differences (FDR≤0.01) between the three *Moricandia* species (Mm= *M. moricandioides*; Ma= *M.arvesnis* line MOR1; Ms= *M.suffruticosa*).
